# Isoegomaketone Alleviates the Development of Collagen Antibody-Induced Arthritis in Male Balb/c Mice

**DOI:** 10.3390/molecules22071209

**Published:** 2017-07-19

**Authors:** Chang Hyun Jin, Yangkang So, Bomi Nam, Sung Nim Han, Jin-Baek Kim

**Affiliations:** 1 Advanced Radiation Technology Institute, Korea Atomic Energy Research Institute, Jeongeup-si, Jeollabuk-do 56212, Korea; chjin@kaeri.re.kr (C.H.J.); yangkang@kaeri.re.kr (Y.S.); bomi1201@kaeri.re.kr (B.N.); 2 Department of Food and Nutrition, College of Human Ecology, Seoul National University, 1 Gwanak-ro, Gwanak-gu, Seoul 08826, Korea; snhan@snu.ac.kr

**Keywords:** isoegomaketone, collagen antibody-induced arthritis, inflammation, rheumatoid arthritis, neutrophil to lymphocyte ratio

## Abstract

In this study, we attempted to identify and assess effects of isoegomaketone (IK) isolated from *Perilla frutescens* var. *crispa* on the development of rheumatoid arthritis (RA). RA was induced in male Balb/c mice by collagen antibody injection. Experimental animals were randomly divided into five groups: normal, collagen antibody-induced arthritis (CAIA), CAIA + IK (5 mg/kg/day), CAIA + IK (10 mg/kg/day), and CAIA + apigenin (16 mg/kg/day) and respective treatments were administered via oral gavage once per day for four days. Mice treated with IK (10 mg/kg/day) developed less severe arthritis than the control CAIA mice. Arthritic score, paw volume, and paw thickness were less significant compared to the control CAIA mice at day seven (73%, 15%, and 14% lower, respectively). Furthermore, histopathological examination of ankle for inflammation showed that infiltration of inflammatory cells and edema formation were reduced by IK treatment. Similarly, neutrophil to lymphocyte ratio (NLR) in whole blood was lower in mice treated with IK (10 mg/kg/day) by 85% when compared to CAIA mice. Taken together, treatment with IK delays the onset of the arthritis and alleviates the manifestations of arthritis in CAIA mice.

## 1. Introduction

Rheumatoid arthritis (RA) is a systemic autoimmune disease in which chronic joint inflammation leads to cartilage destruction and bone erosion [1]. In addition, about 1% of US population is affected by RA, and RA increases the risk for cardiovascular disease, lymphoma, and death [2]. Typically, RA is treated with steroidal/nonsteroidal anti-inflammatory drugs (NSAID) or biological modulators such as tumor necrosis factor alpha (TNF-α) inhibitors and interleutkin-1 (IL-1) receptor antagonists [3]. Acetaminophen, a kind of NSAID, is most frequently implemented and taken in very high doses (4 g/day) [4]. However, the use of standard drugs in RA is known to produce a variety of side effects: Infusion hypersensitivity reactions with the use of TNF-α inhibitors [5]; gastrointestinal ulcerations and hemorrhagic events triggered by NSAID [6]; higher risk of infection due to the use of biological drugs [7]; etc. Therefore, the need for new cure in RA is still high.

Because RA arises from complex etiology, different animal models are implemented to assess the efficacy of new therapies. Collagen-induced arthritis (CIA) is widely used to study RA, and shares many histopathological features of the human arthritis [8]. However, the susceptibility for CIA is low in Balb/c mice and long period of time is required for the induction of arthritis. Collagen antibody-induced arthritis (CAIA) represents a relevant model for studying the efferent phase of RA, where leukocytes are attracted and respond to the focal immune complex in the joint [9]. In the case of CAIA, induction is rapid and results in a steady and controlled disease progression that exhibits histological similarities to the CIA model. 

Isoegomaketone (IK), an essential oil component in *P. frutescens*, exhibits numerous biological properties. Previously, our group isolated IK from radiation-induced mutant *P. frutescens* var. *crispa* [10], and showed that IK reduced NO production and iNOS protein levels through the heme oxygenase-1 (HO-1) induction and inhibition of the interferon-β-STAT-1 pathway in lipopolysaccharide (LPS)-stimulated RAW264.7 cells [11]. Furthermore, IK suppressed NO production in LPS-treated Balb/c mice [11]. Although there is strong evidence to suggest that IK has anti-inflammatory properties, the efficacy of IK as a treatment option for inflammatory disease (such as RA) has not been explored or tested. Therefore, the purpose of the present study was to observe and evaluate the effect of IK on RA in CAIA animal model.

## 2. Results

### 2.1. Effect of IK Treatment on the Development of RA in CAIA Model

**Figure 1:**
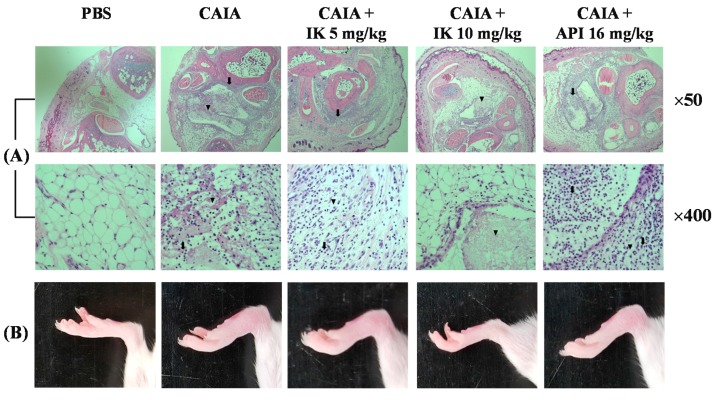
Image of representative microscopic features of knee joint (**A**) and mice joint (**B**). Isoegomaketone (IK) and apigenin (API) were administered via oral gavage once per day for 4 days. Arrow indicates infiltration of neutrophils and arrowhead indicates the necrosis.

**Figure 2:**
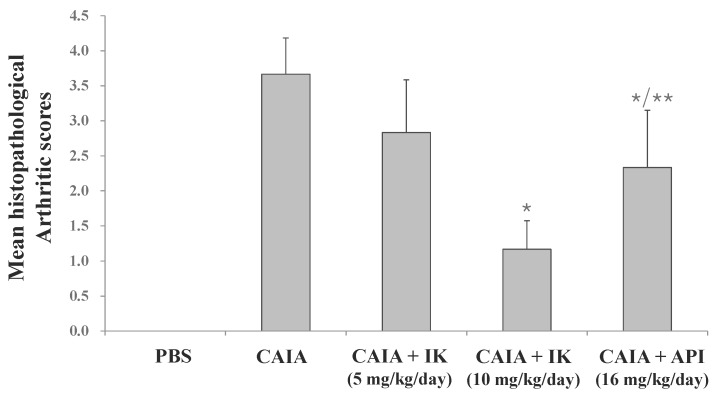
Effect of IK and API on mean histopathological arthritis scores in CAIA mice. Results were expressed as a score (means ± SD) of six mice. * *p* < 0.05 vs. CAIA-group and ** *p* < 0.05 vs. IK(10 mg/kg)-group.

First, in whether IK treatment by oral administration could serve to obviate inception of the disease in Balb/c mice with CAIA was investigated. IK-treated mice developed less severe arthritis in a dose-dependent manner (Figure 1). Both redness and swelling of joints were induced in CAIA group, but those arthritic symptoms were significantly attenuated in IK-treated group (10 mg/kg). Furthermore, IK-treated group (10 mg/kg) showed less severe redness and swelling than the apigenin (API)-treated group. API is one of the bioactive components in plant flavones containing anti-inflammatory activities [12]. Histopathological examinations also indicated that IK treatment reduced synovial hyperplasia, as well as infiltration of inflammatory cells in the joint space (Figure 1). Mean histopathological arthritic score of CAIA-group, IK-treated group (5 mg/kg), IK-treated group (10 mg/kg) and API-treated group were 3.67 ± 0.52, 2.83 ± 0.75, 1.17 ± 0.41, and 2.33 ± 0.82, respectively (Figure 2). Mean histopathological arthritic score of IK-treated group (10 mg/kg) was lower compared with CAIA-group and API-treated group (Figure 2).

### 2.2. Effect of IK Treatment on Paw Volume in CAIA Model

**Figure 3:**
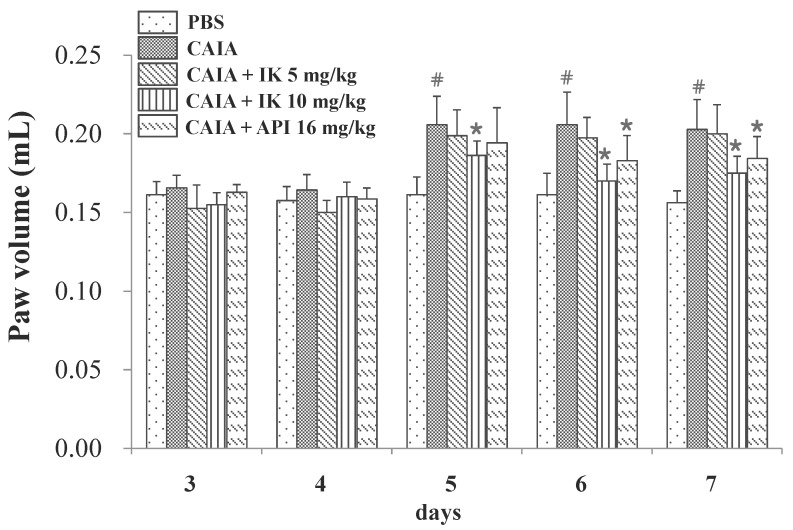
Effect of IK and API on paw volume in CAIA mice. Paw volume were measured using a Digital Plethysmometer every day after lipopolysaccharide (LPS) injection and oral administration of treatments. The average volume of both hind legs was used. Data are presented as means ± SD (*n* = 6). # *p* < 0.05 vs. PBS-group and * *p* < 0.05 vs. CAIA-group.

In an effort to assess any potential effect of IK on the progression of RA in CAIA model, male Balb/c mice were provided with the phosphate buffer saline (PBS) with or without IK from day three through day six. CAIA group showed a significant increase in the paw volume at the days five, six, and seven (27.6%, 27.6% and 29.8%, respectively) compared to the PBS group (Figure 3). Paw volume was significantly lower in the IK-treated group (10 mg/kg), when compared with the control CAIA group on day five, six and seven (9.5%, 17.4% and 13.7%, respectively). Therefore, oral administration of IK seems to attenuate the increase of paw volume in CAIA model. However, IK-treatment at 5 mg/kg didn’t result in any appreciable difference in paw volume, when the treated group was compared with the control CAIA group.

### 2.3. Effect of IK Treatment on Paw Thickness in CAIA Model

**Figure 4:**
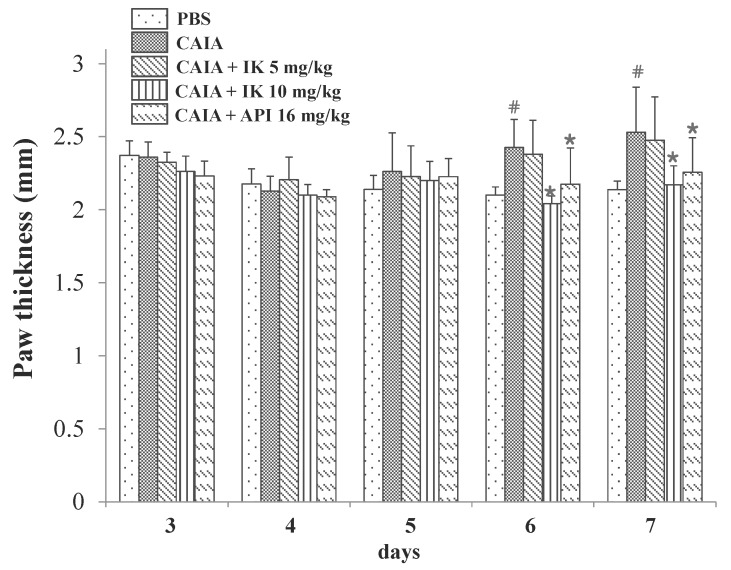
Effect of IK and API on paw thickness in CAIA mice. Paw thickness was measured using a digital caliper every day after LPS injection and oral administration of treatments. The average thickness of both hind legs was used. Data are presented as means ± SD (*n* = 6). # *p* < 0.05 vs. PBS-group and * *p* < 0.05 vs. CAIA-group.

In an effort to assess whether IK had any palpable effect on the progression of RA in CAIA model, paw thickness also was measured by digital caliper. CAIA group showed a significant increase in the paw thickness at days six and seven (15.5% and 18.4%, respectively) compared to the PBS group (Figure 4). Paw thickness was significantly lower in the IK-treated group (10 mg/kg) compared with the control CAIA group at days six and seven (15.8% and 14.2%, respectively). Therefore, oral administration of IK seemed to attenuate the increase of paw thickness in CAIA model. However, IK-treatment at 5 mg/kg didn’t result in significant difference in paw thickness compared with the control CAIA group.

### 2.4. Effects of IK Treatment on Arthritic Score in CAIA Model 

**Figure 5:**
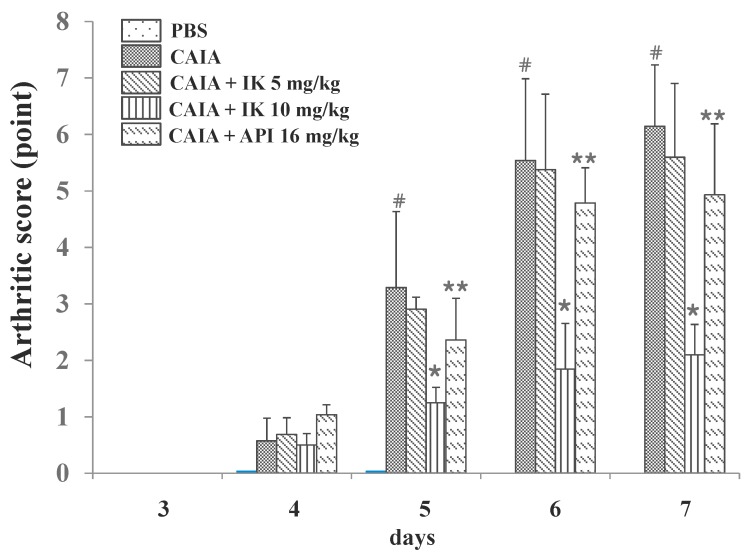
Effect of IK and API on arthritic score in CAIA mice. Arthritic score was done blindly by using a system based on the number of inflamed joints in front and hind paws, inflammation being defined by swelling and redness at the scale from 0 (no redness and swelling) to 3 (severe swelling with joint rigidity or deformity; maximal score for four paws, 12). Data are presented as means ± SD (*n* = 6). # *p* < 0.05 vs. PBS-group, * *p* < 0.05 vs. CAIA-group, and ** *p* < 0.05 vs. IK(10 mg/kg)-group.

Arthritic score was measured (blindly) by four different people in an effort to further determine whether IK suppressed RA progression in CAIA model. CAIA group showed a significant increase in arthritic score from days four through seven compared with the PBS group (Figure 5). The normal group didn’t show any redness or swelling of joints until day seven, however the control CAIA group showed arthritic signs and symptoms in all joints from days four through seven. Those arthritic signs were significantly attenuated in IK-treated group (10 mg/kg) from days five through seven. Oral administration of IK alleviated the arthritic signs such as redness and swelling of joints in CAIA model. Furthermore, IK-treated group (10 mg/kg) showed delayed onset of the signs when the IK-treated group was compared with API-treated group. However, IK-treatment at 5 mg/kg didn’t show significant benefit with regard to the arthritic score, when compared to the control CAIA group.

### 2.5. Effects of IK Treatment on Blood Cell Population in CAIA Model 

**Figure 6:**
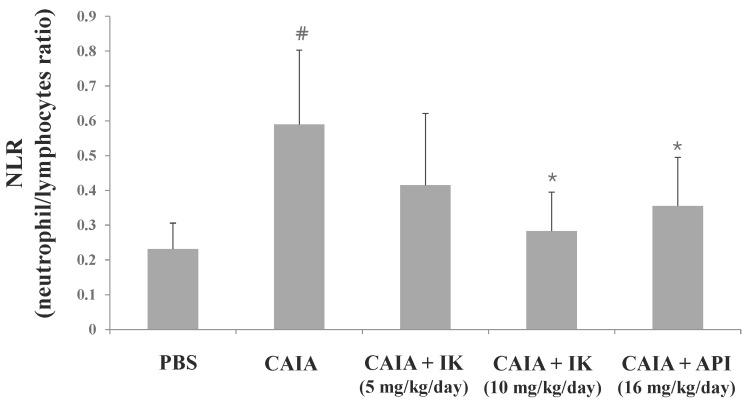
Effect of IK and API on neutrophil-to-lymphocyte ratio in CAIA mice. Whole blood samples were collected by cardiac puncture. Data are presented as means ± SD (*n* = 6). # *p* < 0.05 vs. normal-group and * *p* < 0.05 vs. CAIA-group.

The neutrophil-to-lymphocyte ratio (NLR) is defined as “the proportion of absolute neutrophil count to lymphocyte count in whole blood cells”. It is generally accepted that NLR is a useful marker for the evaluation of inflammatory activity in chronic inflammatory diseases such as ulcerative colitis [13], prostate cancer [14], and RA [15]. To further investigate whether IK affects blood cell population in CAIA model, NLR was measured from the whole blood sample. CAIA group showed a significant increase in NLR at day seven compared to the PBS group (Figure 6). NLR level was lower in IK-treated group (10 mg/kg) than the control CAIA group by 51.9%. However, IK-treated group (5 mg/kg) didn’t show significant result compared with the CAIA group in NLR levels.

## 3. Discussion

In the present study, we evaluated the anti-arthritic effect of IK and compared with the effect of apigenin (API) on the development of CAIA model. The treatment with IK attenuated the infiltration of immune cells into joint synovium, paw edema, arthritic score, and NLR levels. Furthermore, IK treatment showed more effective anti-arthritic activity compared with API in mean histopathological score and arthritic score. At day seven of CAIA, marked infiltration of inflammatory cells into the synovium and cartilage damage were observed. Immune complex in CAIA activates metalloproteinases that cleave collagen and, in turn, induces cartilage matrix loss. From day three, paw edema manifest as volume and thickness increased in CAIA group. However, introduction of IK therapy markedly reduced clinical arthritic scores and the incidence of clinically evident signs and symptoms and additionally, the therapy seems to have blocked synovial inflammation and erosive joint destruction. The results indicate that IK treatment not only obviates the onset of CAIA, but may also decrease the signs and symptoms, the severity, of the disease.

RA is a chronic inflammatory auto-immune disease normally treated by pharmacologic and non-pharmacologic therapies. The pharmacological treatment of RA aims to prevent further development of the disease by introducing anti-rheumatoid drugs in the early phase of the disease [16]. However, significant side effects emanate from these treatments (in the later stages of the disease). Many previous studies suggest that nutrient supplementation has the potential for improving RA. These benefits are achieved by attenuating symptoms and slowing the progression of the RA pathology, as well as obviating potential negative side effects arising from pharmacologic therapy [17]. The efficacy of nutrient supplementation is based on phytochemicals such as polyphenol, flavonoid, tannin, anthocyanin, and glycoside. There are many reports about dietary phytochemicals in RA such as curcumin [18,19], epigallocatechin-3-gallate (EGCG) [20,21], gallic acid [22,23] and resveratrol [24,25].

API is a dietary flavonoid found in fruits, vegetables, and herbs. Many studies have been reported that API has anti-arthritic properties: Suppression of the collagenase activity involved in RA [26]; protection against CIA [12]; induction of apoptosis in rheumatoid fibroblast-like synoviocytes by reactive oxygen species (ROS) and activation of ERK1/2 [27]. Unlike previous study [12] which API was administered intraperitoneally (20 mg/kg), API was administered by oral gavage (16 mg/kg) in this study. In this study, API was used as positive control because it is a well-known anti-arthritic component in *P. frutescens*.

IK is an essential oil component in *P. frutescens*. In previous reports, we already examined various pharmacological activities of IK: anti-inflammatory activities in RAW264.7 cells [11]; anti-cancer activities in human DLD1 cells [28]; anti-obesity activities in 3T3-L1 cells and C57BL/6J mice [29]; and anti-oxidant activities in RAW264.7 cells [30]. To our knowledge, this is the first report to the effect that that IK has real, actual, material and palpable anti-arthritic effect in CAIA animal model. The results of this study encourage the therapeutic use of IK or *P. frutescens* var. *crispa* in the chronic inflammatory situation like RA.

Because RA is caused by complex etiology, different animal models are utilized to evaluate the efficacy of any new therapy. CIA has been widely used to study RA and shares many histopathological features of the human counterpart [8]. CAIA is a relevant model for studying the efferent phase of RA that is mainly mediated by the innate immune system, where leukocytes are attracted and respond to the immune complex in the joint [9]. One particular benefit of CAIA over CIA is that the model is amenable for use in strains not suitable for CIA [31]. Furthermore, CAIA is a fast model and has a high degree of synchronicity in disease onset [31]. In a previous study, IK had anti-inflammatory activities in LPS-treated Balb/c mice [11] that had low susceptibility for CIA. And male mice generally developed stronger arthritic symptoms and had a higher incidence of arthritis development [31]. For these reasons, we induced RA in male Balb/c mice using CAIA for investigating anti-arthritic activity of IK.

## 4. Materials and Methods

### 4.1. Animals

Animals were maintained in accordance with the guidelines of the Guide for the Care and Use of Laboratory Animals (Institute of Laboratory Animal Resources, KAERI-IACUC-2016-017). Male Balb/c mice (four weeks) were purchased from Orient Bio Inc. (Seongnam, Korea) and allowed to acclimate for one week prior to the beginning of the study. Mice were maintained in a room which controlled light/dark cycle (12 h/12 h), temperature (about 23 ± 2°C), and humidity (55 ± 10%).

### 4.2. Sample Preparation 

IK and apigenin (API) was diffused into sterile phosphate-buffered saline (PBS, pH 7.4) containing 0.5% Tween 20 by sonication.

### 4.3. Collagen Antibody-Induced Arthritis

Mice were randomly divided into five groups; (1) PBS (*n* = 6), (2) CAIA (*n* = 6), (3) CAIA plus IK (5 mg/kg, *n* = 6), (4) CAIA plus IK (10 mg/kg, *n* = 6), (5) CAIA plus API (16 mg/kg, *n* = 6). A cocktail of four monoclonal antibodies to type II collagen (ArthritoMab; MD Bioscience, Saint Paul, MN, USA; 2 mg/100 μL) was injected intravenously at day 0. Mice in PBS group were injected with equal volume of sterile PBS. At day three, all animals except PBS group were intraperitoneally injected with LPS (Escherichia coli 055:B5; MD Biosciences; 50 μg/200 μL endotoxin-free water). And treatments (PBS, IK, and API) were administered by oral gavage once a day from day three through day six. Mice were examined for the development of arthritis for four days after LPS injection.

### 4.4. Assessment of Clinical Signs of Inflammation 

Paw volumes were measured using a Digital Plethysmometer (LE7500, Panlab, Spain) every day after LPS injection. The hind leg was soaked in the buffer calibrated with 1 mL standard sinker. The increased volume was measured. The average volume of both hind legs was used. Paw thickness was measured using a digital caliper (Mitutoyo, Andover, UK) every day after LPS injection. The average thickness of both hind legs was used. Arthritic score was done blindly by using a system based on the number of inflamed joints in front and hind paws, inflammation being defined by swelling and redness at the scale from 0 (no redness and swelling) to 3 (severe swelling with joint rigidity or deformity; maximal score for four paws, 12).

### 4.5. Histopathological Assessement

Hind feet were removed after euthanization and fixed using 4.5% buffered formalin. Hind feet were decalcified in buffered formalin containing 5.5% ethylenediaminetetraacetic acid (EDTA). Upon decalcification, paws were embedded in paraffin wax blocks, sectioned, and stained with haematoxylin and eosin for microscopic evaluation, which was performed by an expert blinded to the treatments received. Each section was screened for infiltration of neutrophils to synovium and every joint was scored as follows: 0, normal; 1, minimal; 2, mild; 3, moderate; and 4, marked.

### 4.6. Analysis of Neutrophil and Lymphocyte

Whole blood samples were collected by cardiac puncture. The blood was placed in Vacutainer TM tubes containing EDTA (BD science, Franklin Lakes, NJ, USA). Anti-coagulated blood was used for the determination of the blood cell population analysis including neutrophil and lymphocytes in a HEMAVET 950 (Drew Scientific Inc., Miami Lakes, FL, USA).

### 4.7. Statistical Analysis

One-way analysis of variance (ANOVA) was used to determine overall differences among groups, followed by Fisher’s least significant difference (LSD) analysis for individual group comparisons. The results from all comparisons were considered significant at *p* < 0.05. Data were reported as mean ± SD. All data were analyzed using the SPSS 21.0 program (SPSS Inc., IL, USA).

## 5. Conclusions 

In this study, anti-arthritic activities of IK were investigated in CAIA model. IK treatment delayed the onset and reduced the severity of arthritis in CAIA mice. Therefore, IK has potential for therapeutic use in the chronic inflammatory diseases such as RA.
